# Maximum toe flexor muscle strength and quantitative analysis of human plantar intrinsic and extrinsic muscles by a magnetic resonance imaging technique

**DOI:** 10.1186/1757-1146-7-26

**Published:** 2014-05-05

**Authors:** Toshiyuki Kurihara, Junichiro Yamauchi, Mitsuo Otsuka, Nobuaki Tottori, Takeshi Hashimoto, Tadao Isaka

**Affiliations:** 1 Department of Sport and Health Science, Ritsumeikan University, 1-1-1 Noji Higashi, Kusatsu, Shiga 525-8577, Japan; 2 Future Institute for Sport Sciences, Tokyo, Japan; 3 Graduate School of Human Health Sciences, Tokyo Metropolitan University, 1-1 Minami-Osawa, Hachioji-shi, Tokyo 192-0397, Japan; 4 Faculty of Associated Medical Sciences, Khon Kaen University, Khon Kaen, Thailand

**Keywords:** Toe grip dynamometer, CSA of muscles, Maximum isometric force, Specific force, Imaging technique

## Abstract

**Background:**

The aims of this study were to investigate the relationships between the maximum isometric toe flexor muscle strength (TFS) and cross-sectional area (CSA) of the plantar intrinsic and extrinsic muscles and to identify the major determinant of maximum TFS among CSA of the plantar intrinsic and extrinsic muscles.

**Methods:**

Twenty six young healthy participants (14 men, 12 women; age, 20.4 ± 1.6 years) volunteered for the study. TFS was measured by a specific designed dynamometer, and CSA of plantar intrinsic and extrinsic muscles were measured using magnetic resonance imaging (MRI). To measure TFS, seated participants optimally gripped the bar with their toes and exerted maximum force on the dynamometer. For each participant, the highest force produced among three trials was used for further analysis. To measure CSA_,_ serial T1-weighted images were acquired.

**Results:**

TFS was significantly correlated with CSA of the plantar intrinsic and extrinsic muscles. Stepwise multiple linear regression analyses identified that the major determinant of TFS was CSA of medial parts of plantar intrinsic muscles (flexor hallucis brevis, flexor digitorum brevis, quadratus plantae, lumbricals and abductor hallucis). There was no significant difference between men and women in TFS/CSA.

**Conclusions:**

CSA of the plantar intrinsic and extrinsic muscles is one of important factors for determining the maximum TFS in humans.

## Background

The toe flexor muscle strength (TFS) is one of important essentials to provide postural control in standing and walking [1,2]. The activation of toe flexor muscles is required for the push-off phase of human walking, as the heel leaves the ground and the dorsiflexion of the metatarsophalangeal (MTP) joint increases [3]. Also, increase in TFS contributes an improvement of physical performance [4]. Conversely, a low level of TFS is associated with a high risk of falls in elderly individuals [5] and impairment of physical performance in athletes [6]. Accordingly, an atrophy of toe flexor muscles or plantar intrinsic muscles in the forefoot has been identified in patients with plantar fasciitis [7]. Therefore, appropriate evaluation of TFS is important to quantify the physical activity in daily living and sports.

A promising method of measuring TFS is using the toe grip dynamometer as the direct method [8,9]. This method measures the potential force produced from both plantar intrinsic and extrinsic muscles because these plantar muscles can generate force at the MTP and Interphalangeal joints [8]. Indirect methods are able to estimate TFS by measuring the cross sectional area (CSA) of toe flexor muscles using imaging modalities such as magnetic resonance imaging (MRI) or ultrasound [10,11]. One study estimates TFS at MTP joint using MRI [10]; however, this study may incorrectly estimate TFS. According to Fukunaga et al. [12], flexor hallucis longus (FHL) and flexor digitorum longus (FDL) have the maximum anatomical cross sectional area (ACSA) of 4.85 cm^2^ and 1.59 cm^2^, and physiological cross sectional area (PCSA) of 19.32 cm^2^ and 9.12 cm^2^, respectively. These data suggest the contribution of these muscles to TFS is not ignorable. Therefore, the contribution of both the plantar intrinsic and extrinsic muscles should include estimating TFS.

The force-generating capacity of a muscle is determined by CSA of the muscle, and the muscle force normalized by muscle size or CSA (specific force) can quantify muscle functions involved in human movements [13,14]. A relationship between force generation and CSA of muscle in the lower leg has been reported in dorsiflexor and plantarflexor muscles of the ankle [15,16]; however, there is no information available regarding how TFS is related to CSA of plantar intrinsic and extrinsic muscles. Therefore, the aims of this study were to investigate the relationships between the maximum isometric TFS and CSA of the plantar intrinsic and extrinsic muscles and to identify the major determinant of maximum TFS among CSA of the plantar intrinsic and extrinsic muscles.

## Methods

### Participants

Twenty six young healthy sedentary volunteers (14 men, 12 women; age, 20.4 ± 1.6 yrs; height, 167.5 ± 7.5 cm; body mass, 60.7 ± 8.6 kg; means ± standard deviation [S.D.]) participated in the study. None of them reported any history of diagnosed neuromuscular disorder or lower limb injury. In addition, the participants had no visible symptoms of hallux valgus or toe deformities. Foot anthropological measurements, i.e. foot length (FL) and arch height (AH), were measured using a ruler. FL was determined as the length between the most posterior aspect of the calcaneus and the tip of the longest toe, and AH was defined as the perpendicular distance between the navicular tuberosity and the floor [17]. Next, the arch index (AI) was calculated as the ratio of AH to FL.

The methods and procedures used in this study were in accordance with the current local guidelines and the Declaration of Helsinki, and were approved by the Research Ethics Committee involving Living Human Participants at Ritsumeikan University. All the participants were informed about the study requirements, benefits, and risks prior to study onset. Written consent was obtained from each participant.

### Toe flexor strength (TFS)

**Figure 1:**
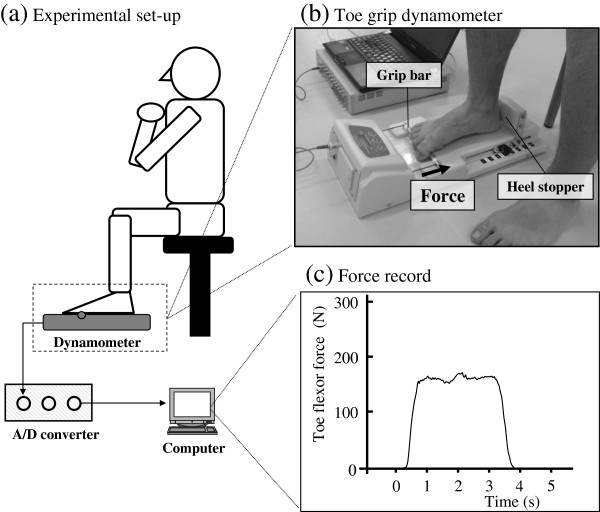
**Measurement for TFS.** The experimental set-up for TFS measurement **(a)**, the toe grip dynamometer **(b)**, and a typical example of the force-time curve during maximum TFS measurements **(c)**. Participants performed their maximum TFS in the sitting position, and they placed their test foot within the heel stopper on the dynamometer. Maximum force was recorded on a computer via A/D converter. During the force measurement, the first proximal phalanx was gripped the grip-bar without pulling a leg.

The maximum voluntary isometric TFS was measured using a specifically designed dynamometer (T.K.K. 3361, Takei Scientific Instrument Co., Niigata), which has a range of force between 1 and 400 N. The experimental setup for maximum TFS measurements is shown in Figure 1. TFS was measured in the sitting condition with 90 degrees of their hip and knee joint angles with the ankle in a neutral position in order to reduce the plantar fascia tension by truss effect. Under vertical loading or body mass on the foot, the plantar aponeurosis was extended by the truss structure of the foot [18]. Participants were instructed to place their test foot within the heel stopper and optimally gripped the grip-bar with their toes on the dynamometer. During the measurements, participants placed their arms in front of their chest and were instructed to perform the task without moving their trunk from the seat or pulling the grip-bar by flexing the knee. Participants first performed a few test contractions as submaximum efforts to familiarize themselves with the measurement. Then, they performed voluntary isometric contractions as explosively as possible and attempted to maintain the maximum force for 3 seconds. Measurements were repeated three times with at least a one-minute rest period between bouts. Both right and left feet were measured in a randomized order. The largest value among the three trials was used for further analysis.

### Magnetic resonance imaging (MRI)

The participants lay in a supine position on the examination table of a 1.5-T MR system (Signa HDxt, GE Healthcare UK Ltd., Buckinghamshire). Whole foot images were acquired using an ankle coil (HD Knee/Foot coil, GE Healthcare UK Ltd., Buckinghamshire) positioned in the center of magnet. To reduce motion artifacts during image acquisition, the foot and ankle were encased in the coil and stabilized with cushions and Velcro straps so that the ankle was at an angle of 15 degrees of ankle plantarflexion. Serial T1-weighted MR images were acquired from the longitudinal distance between sesamoids (0% foot length) and calcaneal tuberosity (100% foot length) of foot perpendicular to the plantar aspect of the foot, using a fast spin-echo sequence (repetition time = 500 ms, echo time = 16 ms, averages = 3, slice thickness = 4 mm, gap between slices = 0 mm, field of view = 120 × 120 mm, flip angle = 90 degrees, matrix = 512 × 512), according to Chang et al. [7]. The data acquisition time for each foot was approximately 9 min. For lower leg images, legs were placed to parallel to the main magnetic field. Serial T1-weighted axial MR images were acquired from the knee cleft just proximal to the malleoli (repetition time = 600 ms, echo time = 7.7 ms, averages = 2, slice thickness = 10 mm, gap between slices = 0 mm, field of view = 360 × 360 mm, flip angle = 90 deg, matrix = 256 × 256). Due to the length of the legs, images acquisition required two passes. The data acquisition time for each scan was approximately 3 min.

**Figure 2:**
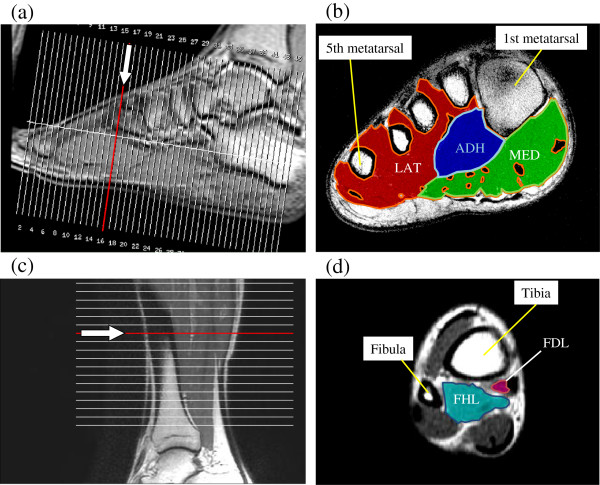
**MRI with user outlined plantar intrinsic and extrinsic muscles group.** A sagittal image of a foot representing the localization of serial axial MRI **(a)**. A typical example of MRI with a manually painted three plantar intrinsic muscle groups **(b)**. A sagittal image of a lower leg representing the localization of serial axial MR images **(c)**. A typical example of the analyzed image for two plantar extrinsic muscles **(d)**. White arrows show the site of the analyzed images. The painted area excludes the bones and the central band of the plantar fascia as well as tendons, fat, connective tissue, nerve tissue and blood vessels.

To determine the location for measuring the CSA, we selected the image at the MTP joint that was near 20% of the longitudinal foot length. ACSA of plantar intrinsic muscle is the largest at the location of 20% of the longitudinal length [7]. Examining each image, we excluded non-contractile tissues such as bone, tendon, fat, connective tissue, nerve tissue and blood vessels, wherever possible. To identify an accurate segmentation of each individual muscle was not possible for the smaller muscles, but anatomical groups of the intrinsic muscles could be identified. Green and Briggs [10] have divided the plantar intrinsic muscles into five groups: medial (abductor hallucis and flexor hallucis brevis), adductor (adductor hallucis), central (flexor digitorum brevis, quadratus plantae and lumbricals), interosseous (dorsal and plantarinterosseous), and lateral (abductor digiti minimi and flexor digit minimi brevis). In this study, the plantar intrinsic muscles were divided into three muscle groups as follows; MED: flexor hallucis brevis, flexor digitorum brevis, quadratus plantae, lumbricals and abductor hallucis, ADH: adductor hallucis, and LAT: abductor digiti minimi, flexor digiti minimi brevis, dorsal and plantar interosseous muscles (Figure 2), because the boundaries of muscle groups were not easily separable to five groups in all subject. For extrinsic foot muscles, FHL and FDL were identified in each image along with the lower limb, and maximum ACSA of each muscle was determined. All measurements and calculations were conducted by the two investigators (TK, NT) using specially designed image analysis software (SliceOmatic 4.3, Tomovision Inc., Montreal).

Using the measured CSA of each muscle, the estimated maximum muscle strength (Fmax) was also calculated from the specific tension. The values of the specific tension were used 15–25 N/cm^2^ for lower and upper estimations of maximum muscle strength [15,16].

### Data analysis

All data were presented in the form of mean ± S.D. The intra-rater reliabilities of TFS measurement were assessed by intra-class correlation coefficient (ICC). A linear regression analysis was performed for the relationship between TFS and CSA of each muscle, and we also examined whether the y-intercept for the regression line was different from zero. Stepwise multiple linear regression models were used to calculate the dependent variables of TFS among test parameters (FL, AH, AI and CSA of each muscle). An unpaired t-test was used to examining the differences between genders. The level of significance was set at p < 0.05.

## Results

TFS was 147.8 ± 55.3 N. The ICCs (1,3) of TFS in right and left foot were 0.960 and 0.931, respectively. Table 1 shows CSA and estimated maximum force of each muscle. TFS/CSA of plantar intrinsic and extrinsic muscle groups was 7.05 ± 1.81 N/cm^2^. FL, AH and AI were 24.4 ± 1.5 cm, 4.8 ± 0.8 cm, and 0.19 ± 0.03, respectively.

**Table 1:** CSA and calculated maximum muscle force of each muscle

	**CSA (cm**^**2**^**)**	**Fmax (N)**
Intrinsic muscle	14.45 ± 3.25	217–361
MED	5.87 ± 1.34	88–147
LAT	6.06 ± 1.58	91–151
ADH	2.51 ± 0.74	38–63
Extrinsic muscle	6.16 ± 1.38	92–154
FHL	5.23 ± 1.17	78–131
FDL	0.93 ± 0.42	14–23

CSA of each plantar intrinsic and extrinsic muscle, and their calculated maximum muscle force (Fmax). All data are expressed as means ± standard deviation (S.D.).

MED: medial parts of plantar intrinsic muscles, LAT: lateral parts of plantar intrinsic muscles, ADH:adductor hallucis muscle, FHL: flexor hallucis longus muscle , and FDL: flexor digitorum longus muscle.

**Figure 3:**
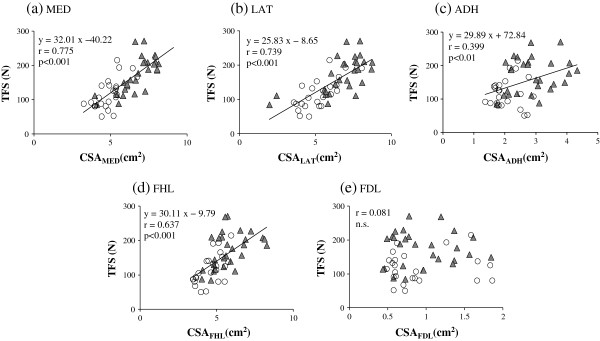
**The relationship between TFS and CSA.** MED: medial parts of plantar intrinsic muscles **(a)**, LAT: lateral parts of plantar intrinsic muscles **(b)**, ADH: adductor hallucis muscle **(c)**, FHL: flexor hallucis longus muscle **(d)**, and FDL: flexor digitorum longus muscle **(e)**. N = 52, triangle: men; open circle: women.

Positive correlations were found between TFS and CSA of all intrinsic and extrinsic muscles, except for FDL (Figure 3), and the y-intercepts of the regression lines between them were not significantly different from zero. Stepwise multiple regression analysis revealed that the determinants of TFS were CSA_MED_ (r = 0.520, p < 0.01) and CSA_LAT_ (r = 0.317, p = 0.040). Other parameters (FL, AH, AI and CSA of other muscles) were not identified as determinants. The stepwise multiple regression equation was obtained as follows:

$$\begin{array}{ll} \mathrm{TFS}\;\left(N\right)= & 21.31\times \left[{\mathrm{CSA}}_{\mathrm{MED}}\left({\mathrm{cm}}^{2}\right)\right]+11.09\\ & \times \left[{\mathrm{CSA}}_{\mathrm{LAT}}\left({\mathrm{cm}}^{2}\right)\right]–44.47 \end{array}$$

In addition, values of TFS (170.5 ± 50.5 N) and sum of CSA of all intrinsic and extrinsic muscles (23.1 ± 3.7 cm^2^) in men were significantly larger as compared with those of TFS (117.8 ± 40.7 N) and CSA (17.7 ± 2.7 cm^2^) in women; however, when TFS was normalized to obtain the ratio TFS/CSA, there was no significant difference between men and women. The values of TFS/CSA for men and women were 7.43 ± 1.68 N/cm^2^ and 6.60 ± 1.89 N/cm^2^, respectively.

## Discussion

This study was the first to show the force-generating capacity of human toe flexor muscles determined by the toe grip dynamometer and MRI in young healthy individuals. The new and important findings from this study were that there were positive relationships between maximum TFS and CSA of both intrinsic and extrinsic muscle groups, and specific force was 7.05 ± 1.81 N/cm^2^. After the stepwise multiple regression analysis, the major determinant of maximum TFS was CSA of medial and lateral parts of plantar intrinsic muscles. Our results suggest that CSA of both intrinsic and extrinsic muscles is important for the maximum force generation of the toe flexor muscles.

TFS was able to directly measure with the toe grip dynamometer and CSA of the plantar intrinsic and extrinsic muscles were able to quantify by using MRI. This measured TFS was positively correlated to CSA of plantar intrinsic and extrinsic muscle groups. Maximum force is related to the size of skeletal muscle [14,19]. CSA of the muscle is the critical factor to determine the muscle force production [15]. Accordingly, greater TFS in men as compare to women reflected to CSA of the foot; however, TFS/CSA was not significantly different between genders. In general, specific tension was not different between individuals [15,16]. Thus, to increase TFS, increases in muscle size of plantar intrinsic and extrinsic muscle are required. Specific exercise training effects of plantar intrinsic and extrinsic muscle are still unknown and further studies should examine the nature of the muscle adaptation in the foot.

FHL and FDL also contributed to TFS because they cross at the ankle joint and MTP joints; however, TFS was not correlated with CSA of FDL. This might be explained by the force-length relationship of muscle. Goldmann and Brüggemann [20] report that flexion moments at MTP is influenced by the ankle joint angle. The fascicle length of FHL muscle changes in 0.5 mm when the ankle rotation is occurred 1 degree [21]. Changes in ankle joint angle are affected to the maximum force level of extrinsic muscles. Thus, FDL may not have an optimum length to produce maximum isometric force at the ankle joint angle of this measurement.

The inherent muscle force-generating capacity is generally determined by specific force. By using MRI, the specific force for ankle plantar flexor and dorsiflexor muscles in vivo has been reported and value is about 15–25 N/cm^2^[15,16,22]. Specific force for toe flexor muscles in this study was somewhat smaller than these values. This might be because all plantar intrinsic and extrinsic muscles were not maximally activated during TFS measurements. Thus, the estimated value of toe flexion strength by product of specific tension (25 N/cm^2^) and muscle volume measured by MRI [10] is overestimated as compared to the value measured by the toe grip dynamometer. The contribution of each muscle in producing TFS is required to determine in the future study by measuring the orientation of the muscles and activation level during force production.

The limitation of this study was that the ACSA of the muscle was measured, yet PCSA was not. For each muscle, PCSA can be calculated using the muscle volume, the fiber pennation angle, and the muscle fiber length [15]. Cadaveric data of pennation angles [23] and fiber lengths [24] of plantar intrinsic muscles are available; however, it was difficult to determine PCSA in this study because most of the major intrinsic foot muscles lie in obliquely, rather than directly, within the transverse or sagittal planes [24], which also hinders MR visibility. With increasing MRI scanning resolution, future studies may be able to quantify PCSA of individual intrinsic foot muscles in vivo for quantifying intrinsic muscle force. Also, future study is required for understanding other factors such as neural drive and contractile properties of muscles of the foot.

## Conclusions

TFS was positively correlated to CSA of plantar intrinsic and extrinsic muscle groups and stepwise multiple linear regression analyses showed that the major determinant of maximum TFS was CSA_MED_. In the future, the contribution of each intrinsic foot muscle during TFS measurements needs to be specified by the muscle activity level of each muscle.

## Abbreviations

ACSA: Anatomical cross-sectional area; ADH: Adductor hallucis muscle; AH: Arch height; AI: Arch index; CSA: Cross-sectional area; FDL: Flexor digitorum longus muscle; FHL: Flexor hallucis longus muscle; FL: Foot length; Fmax: The estimated maximum muscle strength; ICC: Intra-class correlation coefficient; LAT: Lateral parts of plantar intrinsic muscles (abductor digiti minimi, flexor digiti minimi brevis, dorsal and plantar interosseous muscles); MED: Medial parts of plantar intrinsic muscles (flexor hallucis brevis, flexor digitorum brevis, quadratus plantae, lumbricals and abductor hallucis); MRI: Magnetic resonance imaging; MTP: Metatarsophalangeal; PCSA: Physiological cross sectional area; TFS: Toe flexor strength.

## Competing interests

All the authors have no competing of interest to disclose.

## Authors’ contributions

TK and JY conceived and designed the experiments. TK, JY, MO, and NT performed the experiments and analyzed the data. TK, JY and TI contributed materials/analysis tools. TK, JY, MO, TH, and TI drafted the manuscript. All authors read and approved the final manuscript.
